# Supplementary material for: Structural characterization of an ionic liquid in bulk and in nano-confined environment using data from MD simulations
Source: Data Brief. 2019 Nov 23;28:104794. doi: 10.1016/j.dib.2019.104794 (PMC6909096; doi:10.1016/j.dib.2019.104794)
Supplement: Multimedia component 1 [file mmc1.pdf]

Insights from Molecular Dynamics Simulations on Structural Organization and Diffusive Dynamics of an Ionic Liquid at Solid and Vacuum Interfaces

Nataša Vučemilović-Alagić,[a,b] Radha D. Banhatti,[a] Robert Stepić,[a,b] Christian R. Wick,[a,b] Daniel Berger,[c] Mario U. Gaimann,[b] Andreas Baer,[b] Jens Harting,[c] David M. Smith*,[a] and Ana-Sunčana Smith*,[a,b]

[a]Group of Computational Life Sciences, Department of Physical Chemistry, Ruđer Bošković Institute, Bijenička 54, 10000 Zagreb, Croatia

[b]PULS Group, Institute for Theoretical Physics, FAU Erlangen-Nürnberg, Cauerstraße 3, 91058 Erlangen, Germany

[c]Forschungszentrum Jülich GmbH, Helmholtz Institute Erlangen-Nürnberg for Renewable Energy, Fürther Straße 249, 90429 Nürnberg, Germany

Supporting Information

2. Comparative study of force fields

Simple IL - static and dynamic properties

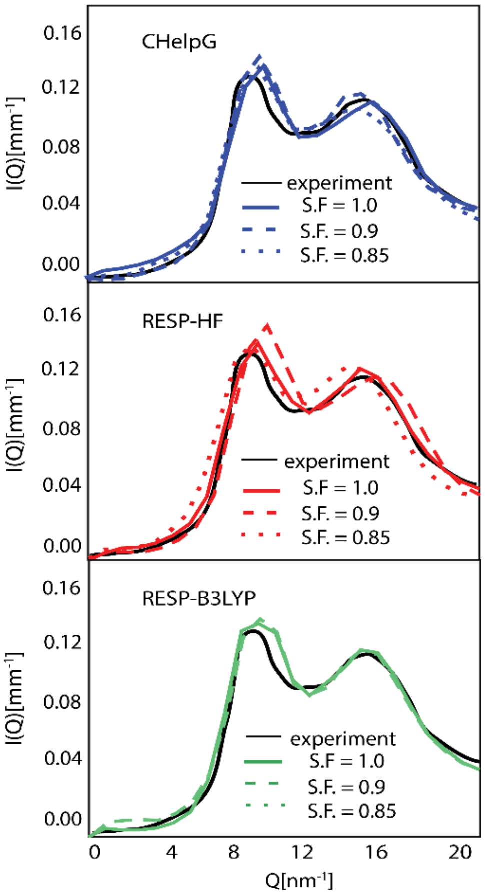

Figure S1. Structure factor (S(q)) as a function q, in pure ionic liquid (L) for all nine parametrization considered in this study, obtained using GROMACS tool based on the Cromer Mann relation from the last 2 ns of production runs. See main text for details. The SWAXS data (momentum range Q of 0.1 to 2 °A^{-1}, with a wavelength λ = 0.75 °A, energy = 16.5 keV) were collected at 25 °C, using a thermostatted bath and a flow-through cell, with proper calibration.[92]

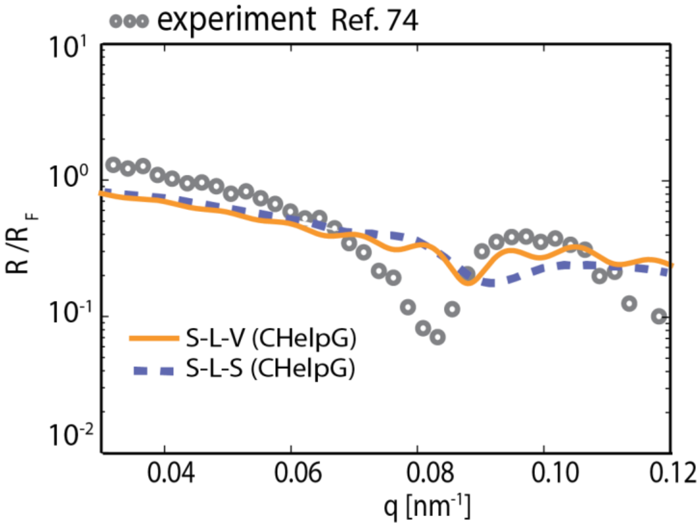

Figure S2. Comparison of the experimental and theoretically calculated normalized X-ray reflectivity curves as a function of momentum transfer q, for the charge method CHelpG with S.F. = 1.0. Orange cure corresponds to the S-L-V system, and purple dashed curve to the S-L-S system in Ref. 74. Experimental data is taken from Ref. 74.

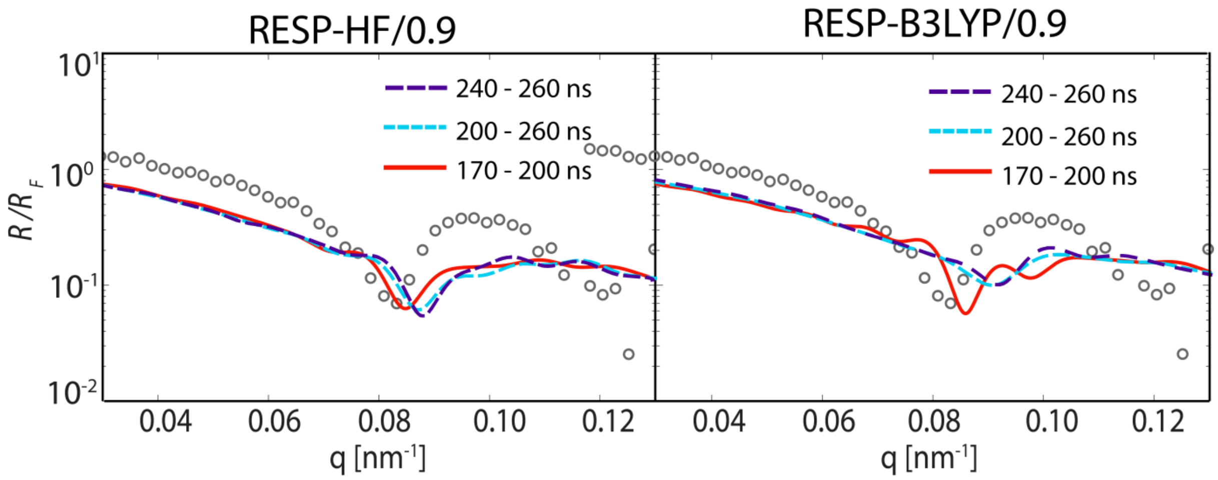

Figure S3. Comparison of the experimental and theoretically calculated normalized X-ray reflectivity curves as a function of momentum transfer q, for the charge methods RESP-HF and RESP-B3LYP with S.F. = 0.9, for three different time windows. The red solid line corresponds to the red dashed line of Fig. 3 of the main text.

References:

74: Brkljača, Z.; Klimczak, M.; Miličević, Z.; Weisser, M.; Taccarrdi, N.; Wasserscheid, P.; Smith, D.M.; Magerl, A.; Smith, A.-S. Complementary Molecular Dynamics and X-ray Reflectivity Study of an Imidazolium-Based Ionic Liquid at a Neutral Sapphire Interface. *J. Phys. Chem. Lett.* **2015**, *6*, 549-555.

92: Russina, O.; Triolo, A.; Gontrani, L.; Caminiti, R.; Xiao, D.; Hines Jr, L. G.; Bartsch, R. A.; Quitevis, E. L.; Plechkova, N.; Seddon, K. R. Morphology and intermolecular dynamics of 1-alkyl-3-methylimidazolium bis {(trifluoromethane) sulfonyl} amide ionic liquids: structural and dynamic evidence of nanoscale segregation. *J. Phys. Condens. Matter* **2009**, *21*, 424121.
